# Benefits and Feasibility of Using Videos to Assess Medical School Applicants’ Empathetic Abilities in Multiple Mini Interviews

**DOI:** 10.1007/s40670-020-01163-0

**Published:** 2020-11-23

**Authors:** Kyong-Jee Kim, Nam Young Lee, Bum Sun Kwon

**Affiliations:** 1 Department of Medical Education, Dongguk University School of Medicine, Gyeongju, South Korea; 2 Department of Psychiatry, Dongguk University School of Medicine, Goyang, South Korea; 3 Department of Physical & Rehabilitation Medicine, Dongguk University School of Medicine, Goyang, South Korea

**Keywords:** Student selection, MMI, Empathy, Technology-enhanced assessment

## Abstract

**Purpose:**

We sought to evaluate the feasibility and benefits of using video-based scenarios in Multiple Mini Interviews (MMIs) to assess candidate’s empathic abilities by investigating candidate perceptions and the acceptability, fairness, reliability, and validity of the test.

**Methods:**

The study sample was candidates for admission interviews held in the MMI format at a medical school in South Korea. In this six-station MMI, one station included a 2-min video clip of a patient-doctor communication scenario to assess candidate emphatic abilities, whereas paper-based scenarios were used in the other stations. Candidate’s perceptions and acceptability of using the video-based scenario in the empathy station were examined using a 41-item post-MMI questionnaire. Fairness of the test was assessed by means of differences in candidate perceptions and performance across different demographics or backgrounds. Construct validity was assessed by examining the relationship of candidate performances in the empathy station with those in other stations. The G-coefficient was analyzed to estimate the reliability of the test.

**Results:**

Eighty-two questionnaires were returned, a 97.6% response rate. Candidates showed overall positive perceptions of the video-based scenario and they found it authentic and interesting. The test was fair as there were no differences in candidates’ perceptions of the patient-doctor relationship presented in the video clip and neither in their performance nor in their perceived difficulty of the station across demographics or backgrounds. Construct validity was established as candidate performance in the empathy station was not associated with that of any other stations. The G-coefficient was 0.74.

**Conclusions:**

The present study demonstrates that the video-based scenario is a feasible tool to assess candidate’s empathy in the MMI.

## Introduction

Student selection is an important step in educating tomorrow’s doctors, and during this process, it is critical that candidates’ cognitive and non-cognitive attributes be assessed in order to select those with the qualities required of good doctors [1]. Several research studies have shown the Multiple Mini Interview (MMI) is a valid and reliable tool for assessing candidates’ non-cognitive qualities in medical student selection [2–17]. Meanwhile, it has also been reported that the reliabilities of MMIs can vary widely depending on the way they are administered and structured [2, 6], which suggests research is needed to determine effective formats for MMIs.

Although MMIs are widely recognized as a selection tool in medical education, our review of relevant literature indicates little attention has been paid to using technology in MMIs. There has been a plethora of research and practice on using technology in medical education to improve student learning and assessment [18, 19]. Moreover, technology is increasingly incorporated into medical school admissions processes [20], which include using video-based scenarios in situational judgement tests [21]. Furthermore, with the COVID-19 pandemic rapidly changing the landscape of medical education, it is likely that there will be increasing use of technology in medical school admissions processes. Still, research is scant on the use of technology in MMIs. Thus, research is needed on the MMI format that makes effective use of technology.

Candidates’ various non-cognitive attributes have been assessed in MMIs. In particular, empathy, which is “a personality trait that enables one to identify with another's situation, thoughts, or condition by placing oneself in their situation [22],” is an important attribute in doctors, and therefore, many medical schools assess candidates’ basic understanding of empathy at admission interviews [23]. However, research is scant on how to assess empathy for student selection purpose [23]. Therefore, research is warranted on developing a feasible tool to assess candidate’s empathy in the MMI.

In the present study, we examined the feasibility of using a video-based scenario in the MMI to assess candidates’ empathic abilities. In general, MMI scenarios are presented in a paper format, but technological developments offer opportunities to adopt other formats for presenting scenarios during MMIs. In particular, it has been advocated candidate’s non-cognitive attributes need to be assessed in authentic contexts [24]. Consequently, some medical schools have reported using actors or standardized patients (SPs) in MMI stations to assess candidates’ empathy or communication skills [4, 6]. Still, using actors or SPs are resources-intensive, and there is a lack of research that provides empirical evidence on the utility of MMIs that utilize such resources. Therefore, it is worthwhile to explore other feasible alternatives to assess candidate’s empathic abilities in MMIs.

We considered that presenting candidates with a scenario involving human interactions on a video vignette would be likely to provide richer contexts than scenarios presented as text. The use of videos to present cases or problems has been studied in case-based learning (CBL) and problem-based learning (PBL) situations, and these studies have shown video-based CBL and PBL are more effective than using paper-based alternatives in terms of fostering critical thinking and interest [25–29]. Therefore, it is speculated that using video-based scenario in MMIs provides candidates with more authentic contexts and enhances interest and engagement. Thus, this study aimed to investigate the feasibility of the use of video-based scenarios in MMIs to assess candidate’s empathic abilities by investigating its perceived benefits, acceptability, fairness, reliability, and validity.

## Methods

### Study Participants and Setting

This study was conducted on candidates that participated in admission interviews at Dongguk University School of Medicine (DUMS), a private medical school in South Korea, for matriculation in 2019. DUMS has a 4-year basic medical education program for graduate-entry students and an annual intake of approximately 50 students. DUMS has implemented admission interviews for those that passed the initial screening stage based on considerations of prior academic achievements, including undergraduate Grade Points Average (GPA) and performance at the Korean medical school entrance exam (the Medical Education Eligibility Test). As a result, 84 candidates attended admission interviews, which were conducted in December 2018.

DUMS has implemented MMIs for admission interviews since 2014. Interview schedules are composed of six mini-interviews conducted at separate stations, and the allocated time per station is 10 min. There was one assessor for each station, who evaluated the candidates’ performance on a 5-point scale of 1 being “unsuitable” to 5 being “outstanding” on two to three items presented in a scoresheet. Candidates’ performances are assessed by each station score and their overall performance is determined by summing up the scores at each and every station. Previous experiences with the MMI at DUMS have shown that it is a feasible tool for student selection [30, 31].

### Study Design and Procedures

A video-based scenario was developed for one MMI station to assess candidate’s empathic abilities. MMIs at the other five stations were implemented in a traditional paper-based format. Three medical faculty members participated in the development of the video-based scenarios for the empathy station. Two were experts in MMIs and the other was a psychiatrist, who wrote the script for the scenario. Two investigators with experience of MMIs reviewed and revised the draft scenario. The video was produced in-house and was pilot tested on a volunteer medical education graduate student. The student was asked to think aloud what she thought of the video clip as she watched it and reported whether she thought the situation was presented clearly in the video and whether there was any unambiguity in the dialogue.

The video vignette presented a fictive clinical situation where a doctor interviewed a patient who seemed to be in a depressive mood. The vignette lasted around 2 min because of time constraints for the candidate to view and prepare for him/her to discuss it with the assessor. Candidates used a tablet and a headset to watch the video. During the interview, candidates were asked to assess the extent to which the doctor showed empathy and to elicit the feelings and views of the patient shown in the video, which are considered key elements of empathy in doctor-patient interactions [23]. The candidate also discussed with the assessor about the importance of empathic communication in patient-doctor relationship.

Data on candidate perceptions and performance in the MMI stations were obtained and analyzed to investigate its acceptability, fairness, validity, and reliability as evidence regarding the feasibility of the test. Acceptability by candidates and their perceived benefits of the video-based scenario was examined by using a post-MMI questionnaire. Fairness of the test was assessed by means of differences in candidate perceptions of the MMI and of video-based scenario and in their performance in the empathy station across different demographics or backgrounds. Construct validity was assessed by examining the relationship of candidate scores in the empathy station with those in other stations. Moreover, we calculated the G-coefficient to investigate its reliability using the variance components method.

The post-MMI questionnaire used in this study consisted of 41 items with Likert-type responses ranging from “strongly disagree” (1) to “strongly agree” (5). The questionnaire is composed of the following four sections. The first section included 7 items regarding candidate demographics and backgrounds. The second section consisted of 17 items that elicited candidates’ overall perceptions of the MMI. The items in this section were adapted from the instrument developed by Eva et al. [4] and translated into Korean by Kim et al. [31] and have been used in other studies [30, 31]. The third section included 12 statements on respondent perceptions of the video-based scenario used in the empathy station, which consisted of the following four sub-scales: (a) station difficulty (3 items), (b) authenticity (3 items), (c) interest (3 items), and (d) overall satisfaction (3 items). This section also included five items regarding candidate perceptions of the patient-doctor relationship presented in the video clip. The items in this section of the questionnaire were developed by the authors and were pilot tested in the previous year with a sample of medical school applicants. The last item was a single open-ended question that elicited candidates’ overall opinions of the MMI.

The questionnaire was administered during a wrap-up session conducted immediately after all interviews had ended in the morning and afternoon sessions. Participation in the study was voluntary and consent was implied by return of the questionnaire as responses were collected anonymously. An ethical review was conducted and the study was exempted from the requirement for informed consent by the institutional review board of Dongguk University, Gyeongju.

### Data Analysis

Descriptive statistics were used to analyze candidate responses to the post-MMI questionnaire and their test scores in the MMI stations. Reliability of the research instrument was assessed using Cronbach’s alpha coefficients. The independent *t* test was conducted to compare candidates’ responses and performance with respect to gender and age, for which they were dichotomized about median age (25 years), and their geographic locations (urban vs. rural areas). ANOVA (analysis of variance) was used to compare candidates’ perceptions with respect to undergraduate backgrounds, which were categorized into seven groups. The G-coefficient was analyzed to investigate the reliability of this test, which indicates the proportion of variance in MMI score attributable to differences in candidates’ non-cognitive abilities [11]. The data were analyzed using SPSS version 23 for Windows (IBM Corp., Armonk, USA), and statistical significance was accepted for *p* values < 0.05.

## Results

### Candidate Demographics and Backgrounds

A total of 82 questionnaires were returned, which yielded a 97.6% (82/84) response rate. Twenty-six of the respondents (31.7%) were female and 56 (68.3%) were male; their ages ranged from 22 to 36 years (M = 26.6, SD = 2.83). Candidates’ undergraduate backgrounds were as follows: life sciences (*n* = 35), engineering (*n* = 25), sciences (*n* = 15), health-related professions (*n* = 10), social sciences and humanities (*n* = 3), and others (*n* = 4). Thirty-eight (46.3%) of them were from urban areas, whereas 44 (53.7%) were from rural areas.

### Candidate Perceptions of the Video-Based Scenario

Candidates were neutral with regard to whether the empathy station required specialized knowledge (M = 2.83, SD = 1.02) and with respect to station difficulty (M = 3.17, SD = .75). Nine candidates (10.7%) answered the time allocated to prepare responses to the assessor in the empathy station was too short, whereas the remainder (89.3%) thought it adequate.

Table 1 shows the descriptive statistics regarding candidate perceptions of the video-based scenario and the results of the reliability analysis. Candidates disagreed slightly with the statement that it was difficult to understand the situation presented in the video. Candidates agreed with the statements that the video was authentic and interesting and that they were generally satisfied with it. Cronbach’s alpha values of the four sub-scales of candidate perceptions of the video-based scenario demonstrated acceptable internal consistency of the items.

**Table 1:** Descriptive statistics of candidate perceptions of the video-based scenario in the empathy station (*n* = 82)*

Category	Minimum	Maximum	Mean (SD)	Cronbach’s alpha
Station difficulty	1.00	4.00	2.55 (.73)	.75
Authenticity	1.67	5.00	3.96 (.56)	.73
Interest	1.67	5.00	3.89 (.72)	.81
Overall satisfaction	2.33	5.00	4.21 (.58)	.85

*Note: 1 = “strongly disagree”; 5 = “strongly agree”

### Candidate Perceptions of Empathy from the Situation Portrayed in the Video

Table 2 describes candidates’ perceptions of the extent to which the doctor in the video showed empathy for the patient. The candidates generally evaluated that the patient-doctor relationship presented in the video was not effective in terms of emphatic communication.

**Table 2:** Descriptive statistics of candidate perceptions of the patient-doctor relationship presented in the video clip (*n* = 82)*

I felt …	Mean (SD) *
The doctor showed empathy in communicating with the patient.	1.98 (.93)
The doctor responded appropriately to the patient’s thoughts or feelings.	1.95 (.91)
The doctor communicated in ways to make the patient talk openly about her conditions.	2.33 (1.06)
The patient seemed to feel comfortable talking to the doctor about her thoughts and feelings.	2.00 (.86)
Overall, this patient-doctor communication went well.	2.18 (.90)

*Note: 1 = “strongly disagree”; 5 = “strongly agree”

### Comparisons of Candidate Perceptions of and Performances

Table 3 illustrates candidate perceptions of the video-based scenario for the empathy station across different demographics or backgrounds. Candidates did not differ in their overall perceptions, genders, ages, geographic locations, or undergraduate majors. Yet, male candidates were more satisfied with the video-based scenario than females (*p* < .05), and younger candidates showed more interest in it than their older counterparts (*p* < .05). Moreover, there were no differences in candidate perceptions of empathy from the patient-doctor relationship portrayed in the video clip across genders, ages, locations, or with undergraduate majors.

**Table 3:** Comparisons of the perceptions and performances of candidates with different backgrounds in the empathy station (*n* = 82)

Item	*p* values			
	Gender	Age	Undergraduate major	Geographic location
Candidate perceptions of the video-based scenario				
Station difficulty	.12 (*t* = 1.59)	.08 (*t* = 1.79)	.25 (*F* = 1.36)	.16 (*t* = 1.43)
Authenticity	.05(*t* = 2.05)	.27 (*t* = 1.11)	.84 (*F* = .41)	.32 (*t* = 1.00)
Interest	.11(*t* = 1.60)	.04 (*t* = 2.03)	.70 (*F* = .60)	.39 (*t* = .87)
Overall satisfaction	.04(*t* = 2.04)	.46 (*t* = .75)	.87 (*F* = .37)	.34 (*t* = .95)
Total	.13(*t* = 1.53)	.39 (*t* = .86)	.82 (*F* = .44)	.77 (*t* = .29)
Candidate perceptions of the patient-doctor relationship portrayed in the video				
The extent to which doctor showed empathy	.24 (*t* = 1.19)	.24 (*t* = 1.17)	.30 (*F* = .12)	.15 (*t* = 1.46)
Doctor responding to the patient’s feelings adequately	.17 (*t* = 1.37)	.16 (*t* = 1.42)	.15 (*F* = .17)	.45 (*t* = .76)
Doctor having patient talk openly about her conditions	.92 (*t* = 1.00)	.31 (*t* = 1.01)	.42 (*F* = 1.01)	.46 (*t* = .73)
Patient expressing thoughts and feelings comfortably	.58 (*t* = .55)	.44 (*t* = .77)	.16 (*F* = 1.65)	.44 (*t* = .77)
Overall assessment of patient-doctor relationship	.40 (*t* = .85)	.20 (*t* = 1.29)	.53 (*F* = .83)	.11 (*t* = 1.24)
Total	.11 (*t* = 1.63)	.37 (*t* = .88)	.82 (*F* = .44)	.77 (*t* = .29)
Station scores				
Empathy station	.52 (*t* = 1.26)	.78 (*t* = .28)	.55 (*F* = .70)	-
Total scores (six stations)	.53 (*t* = .32)	.18 (*t* = 1.34)	.05 (*F* = 2.52)	-

Candidates’ performances in the MMI are presented in Table 3. Candidates’ performance in the empathy station did not differ across different demographics or backgrounds nor were there differences in their overall test scores.

Table 4 shows the relationship of candidate performance among MMI stations. The candidate performance in the empathy station was not associated with that of any other stations.

**Table 4:** Pearson’s *r* coefficients of the candidate scores in the MMI stations (*p* values)

Station topic	Self-regulation	Problem-solving ability I	Ethics	Problem-solving ability II	Logical reasoning
Empathy station	.167 (.132)	.130 (.242)	.050 (.654)	.100 (.370)	.055 (.620)

### Reliability Analysis

Table 5 shows the results of the reliability of the test using the variance components method. The G-coefficient of MMI scores was 0.74, which is in an acceptable level.

**Table 5:** Summary of effects, estimated variance components, and the G-coefficient

Effect	Degree of freedom	Mean square	Estimated variance
Candidate	83	77.36	1.93
Station	5	1321.93	3.60
Candidate × station	415	49.57	4.03

G-coefficient = σ^2(candidate)^ / (σ^2(candidate) +^ σ^2(candidate × station)^ /6) = 0.74

## Discussion

The present study illustrates the feasibility of using the video-based scenario in MMIs to assess candidates’ empathic abilities by demonstrating the acceptability, fairness, validity, and reliability of the test. This study also found benefits of using video-based scenarios in MMIs as they present scenarios in an authentic way and hold candidates’ interest.

### Acceptability and Perceived Benefits of Video-Based Scenarios

Our study showed the positive acceptability of our new MMI station by the candidates. The candidates reported overall satisfaction with the use of video-based scenario in the MMI and they agreed it was authentic and interesting. Our study demonstrates that there are benefits of using video-based scenarios in MMIs as they present scenarios in an authentic way and hold candidates’ interest. The findings concur with that observed in other studies that showed using video-based cases in assessments for the health professionals enhanced its authenticity [21] and that it has benefits over traditional text-based format in terms of student preference, enhancing cognitive engagement and stimulating interest [25–29].

### Fairness, Reliability, and Validity of the Test

In terms of candidate perceptions of the video-based scenario for the MMI, this study found the male candidates were more satisfied with it than females, and younger candidates showed more interest in it than their older counterparts. This finding may indicate the digital technology preference among younger males [32]. Yet, there were no differences in candidates’ overall perceptions of the video-based scenario used in the MMI nor in their station scores across demographics or backgrounds; thus, such differences seemed not to affect the overall fairness of the test.

The candidates generally evaluated that the patient-doctor relationship presented in the video was not effective in terms of emphatic communication, which was intended in this case scenario. This finding indicates candidates were generally able to identify the level of empathy that the doctor showed in the patient-doctor communication depicted in the video. The candidates did not differ in terms of their interpretations of patient-doctor relationship depicted in the video with respect to age, gender, geographic, or undergraduate backgrounds. Moreover, candidates’ performance at the video-based MMI station and their perceived station difficulty did not differ with respect to their demographics or backgrounds. These findings indicate that this MMI station using a video-based scenario was fair as it was not biased against candidates on the basis of age, gender, undergraduate majors, or geographic locations.

Our study showed the test was reliable in terms of the generalizability theory, which demonstrated a reliable level of G-coefficient. Moreover, candidate performance in the empathy station was not associated with that of any other stations, which indicate this station assessed candidate attributes that were different from other stations. This finding offers evidence for the construct validity of the test.

### Study Limitations and Recommendations for Future Research

Several limitations should be acknowledged. First, the video vignette used in this study was designed to assess candidate’s empathic abilities. It is known that individual’s non-cognitive abilities are context-specific [33], which means the results of this study cannot be generalized to enable assessments of candidates’ attributes in other domains. Thus, we recommend additional studies be undertaken to develop video-based MMI stations in various domains and establish their effectiveness. Second, although there are many psychometric measures available to assess one’s empathic abilities, we could not compare the results from such measures with the test scores from our empathy station due to the anonymity of our study participants. Such a study would offer further evidence for the validity of this MMI station. Third, our study does not provide evidence on the predictive validity of the test. Future study is warranted to investigate relationships between candidate performance measured by the MMI using a video-based scenario and their empathetic communication skills performed in clinical settings. Fourth, the video-based case was used for the student selection purpose in this study, and it is not clear from this study whether our findings are applicable to its use in other teaching and learning contexts. Future research is recommended for using video-based cases in teaching or assessing student empathy to study its utility in various contexts.

## Conclusions

Our findings indicate that the use of a video-based scenario in the MMI to assess candidates’ empathy was perceived positively by the candidates and it fairly assessed their empathetic abilities as it was not biased against specific demographics or backgrounds. Furthermore, the test was found reliable in terms of the generalizability theory and it was valid as it assessed candidate attributes different from those assessed in the other stations. As Kreiter and Alexson [34] suggest, the practice of MMI can improve by research offering valid evidence. This study adds our knowledge base on the evidence of the feasibility of using video-based scenarios in MMIs.

This study demonstrates the benefits and feasibility of using a video-based scenario in MMIs to assess candidates’ non-cognitive attributes such as empathy. We believe that using video-based scenarios is more effective in assessing communication and interpersonal skills than using paper formats in MMIs because they include verbal and non-verbal cues that encompass the natures of interactions, which allow for authentic assessment. Although some medical schools have reported using actors or SPs in the MMI to assess candidates’ interpersonal skills, often budgetary constraints prevent the utilization of such resources. Thus, we would argue using video-based scenarios provides a more cost-effective means of assessing candidates’ non-cognitive attributes in MMIs, especially in the domains of communication and interpersonal skills such as empathy.
